# Predictors of dying at home for patients receiving nursing services in Japan: A retrospective study comparing cancer and non-cancer deaths

**DOI:** 10.1186/1472-684X-10-3

**Published:** 2011-03-03

**Authors:** Sumie Ikezaki, Naoki Ikegami

**Affiliations:** 1 Department of Health Policy & Management, Keio University School of Medicine, Tokyo, Japan

## Abstract

**Background:**

The combined effects of the patient's and the family's preferences for death at home have in determining the actual site of death has not been fully investigated. We explored this issue on patients who had been receiving end-of-life care from Visiting Nurse Stations (VNS). In Japan, it has been the government's policy to promote end-of-life care at home by expanding the use of VNS services.

**Methods:**

A retrospective national survey of a random sample of 2,000 out of the 5,224 VNS was made in January 2005. Questionnaires were mailed to VNS asking the respondents to fill in the questionnaire for each patient who had died either at home or at the hospital from July to December of 2004. Logistic regression analysis was respectively carried out to examine the factors related to dying at home for cancer and non-cancer patients.

**Results:**

We obtained valid responses from 1,016 VNS (50.8%). The total number of patients who had died in the selected period was 4,175 (cancer: 1,664; non-cancer: 2,511). Compared to cancer patients, non-cancer patients were older and had more impairment in activities of daily living (ADL) and cognitive performance, and a longer duration of care. The factor having the greatest impact for dying at home was that of both the patient and the family expressing such preferences [cancer: OR (95% CI) = 57.00 (38.79-83.76); non-cancer: OR (95% CI) = 12.33 (9.51-15.99)]. The Odds ratio was greater compared with cases in which only the family had expressed such a preference and in which only the patient had expressed such a preference. ADL or cognitive impairment and the fact that their physician was based at a clinic, and not at a hospital, had modest effects on dying at home.

**Conclusions:**

Dying at home was more likely when both the patient and the family had expressed such preferences, than when the patient alone or the family alone had done so, in both cancer and non-cancer patients. Health care professionals should try to elicit the patient's and family's preferences on where they would wish to die, following which they should then take appropriate measures to achieve this outcome.

## Background

How end-of-life care should appropriately be provided has been a major policy issue [1-3]. Many factors have to be considered in deciding what is "appropriate", including the patient's condition and preferences [4-6], the situation of the family [6,7], and the services available [8-10]. If the patient prefers to die at home, that should be respected as much as possible, but the patient might not be in a position to express his or her wish, and the family's preference and capacity to provide care must also be taken into consideration [11-13].

Epidemiological surveys using death certificates have shown that the older the patient, the more likely he or she is to die in a non-hospital setting (i.e. nursing home or own home) [14-16]. For patients with activities of daily living (ADL) and cognitive problems in institutional settings, the preferences of the family or the attitude of the nursing home directors may take precedence in determining the place of death [17-19]. For cancer patients, after reviewing the literature focusing on 17 factors associated with dying at home, Gomes concluded that the patient's low functional status, the patient's preferences, the availability of home care and family support were significant factors [20].

We decided to focus on the combined effects of the patient's and the family's preferences in determining the actual site of death in cancer and non-cancer patients.

Both have been noted as being independently related to the site of death [6,21,22], but how they affect each other has only been confirmed by an interview survey [23], and has yet to be statistically analysed.

As subjects, we chose patients who had been receiving professional nursing services in their homes. The reason for doing so is because the Japanese government has announced a policy initiative to promote end-of-life care at home by expanding Visiting Nurse Stations (VNS) services [24,25]. However, visiting nurses have not received formal specialized training in end-of-life care so that whether they could contribute in realizing the policy goal of increasing the proportion of deaths at home has yet to be investigated.

The other reason why we chose to focus on patients served by VNS was because access to death certificate data is denied to researchers in Japan [26]. Thus, information is only available through either the providers' records, or through questionnaires sent to family members of the deceased patients by providers.

Studies have been made using data from VNS but they have been conducted either prior to the implementation of the long-term care insurance (LTCI) [27], or were limited in their scope to cancer patients after its implementation [5,21]. The implementation of the LTCI may have had a significant impact on the possibility of dying at home because it has greatly expanded home and community services, and by doing so, has decreased the burden to the family care giver [6,11].

In Japan, the proportion dying at home has declined to 12.3% of all deaths, while the proportion dying in hospitals had increased to 79.4% [28], which is high compared with 34% in the Netherlands and 58% in England [29]. Developing palliative care services would have been obvious solution but the limited resources have been mostly targeted on palliative inpatient care units [30,31]. These units may only admit patients diagnosed with either cancer or AIDS by health insurance regulations [32], and although their number has increased, the percentage of deaths from cancer that occur in these units is still only 6% [33].

The aims of this study are as follows:

• To describe and compare cancer patients and non-cancer patients who had been receiving VNS service prior to their death at home or in hospital

• To analyse the factors related to dying at home in the two groups, and focusing on the combined effects of the patient's and the family's preferences

## Methods

### Design

This was designed as a retrospective case-control study of patients served by VNS. Of the patients who had died during the period observed, those who had died at home were regarded as the case group, and those who had died in a hospital within four weeks after admission to the hospital were regarded as the control group.

### Sample and participants

At the time of our study, there were 5,224 VNS [34]. Unlike other countries where visiting nurses focus on post-acute care, VNS were first established in 1992 to provide medical supervision, personal hygiene assistance and guidance to families on care giving for bed bound patients on a long-term basis [35]. When the public long-term care insurance (LTCI) was established in 2000, most of their services were transferred from health insurance to the LTCI. However, the service for patients who required more intensive care, such as those with amyotrophic lateral sclerosis and terminal cancer, remained with health insurance. Almost all VNS nurses provide services for both groups of patients, each having her assigned patients. At the time of our survey, the total number of those receiving community LTCI services and health insurance financed VNS services was 2.14 million, of which 0.28 million (13%) were using VNS services [34]. Among those receiving VNS services, it has been reported that three in one was bedbound, and four in one required professional nursing services such as suction, drip infusion and pressure ulcer care [36]. A national report estimated that, among those dying at home, 17% had been receiving VNS services [34].

Our sample was recruited in the following way. First, we selected VNS by random sampling. A list of VNS to be surveyed was made from a national electronic database of 5,224 VNS, from which we selected 2,000 stratified by postal code at regular intervals so as to derive a geographically representative sample (sampling rate: 38.3%). The number of VNS selected was set, with an expected response rate of 50%, so that 1,000 valid responses could be obtained. The number 1,000 has been regarded as being the goal to obtain a nationally representative sample in Japan and for social surveys in general [37].

Next, questionnaires on patients who had died from July to December of 2004 were sent to the VNS selected. This period of six months was chosen after taking into consideration the burden to the VNS. According to Fukui [21], the average number of deaths per VNS was 0.7 deaths per month so that this would require filling out an average of four forms per VNS. Patients under twenty years old were excluded because they differed in terms of disease types, clinical care and family support [38].

### Procedure

Questionnaires were sent to 2,000 VNS in January, 2005. A cover letter explained the objective of the study, with a note stating that the anonymity of the patients and nurses would be strictly preserved. Consent to participate was indicated by the return of the questionnaire by mail.

The VNS nurse in charge of each patient completed the questionnaires, which consisted of one page each for every patient meeting the criteria based on the information in their nursing records [Additional file 1]. All data were returned from the VNS in one lot. Each returned questionnaire was assigned an ID number by two persons other than the authors.

### Ethical consideration

In view of the audit nature of the research, a formal ethics proposal was not required at the time of the study in Japan so that consent was not requested from the bereaved families [39]. The cover letter clearly stated that the decision to participate in our study was a voluntarily one to be made by the director of the VNS. No identifying information was collected or stored. All data were aggregated for analysis.

### Measure

We selected variables that would be uniformly available in the VNS records and have been cited as relating to the site of death in two review articles [7,20]. ADL function was evaluated based on a 4 level score of their mobility: almost independent (J), home bound (A), chair bound (B), completely bed bound (C) [40]. Cognitive function was evaluated based on a 6 level score: completely independent, almost independent having no problem in daily life (I), occasional monitoring (II), daily care assistance (III), continuous daily care assistance (IV), special professional care (M) [40]. The ADL and cognitive status must be recorded in the physician's order form, which is then transcribed into VNS records. These orders are given in the beginning of every calender month, based on reports from the VNS to the doctor. Thus, the condition recorded is that at the beginning of the month when death occurred or when hospitalized, except when the VNS services were provided for less than one month, in which case, the condition when the services had commenced was coded. We dichotomized the ADL function into bed bound (C) and others [5,21] because, in the former, patients and families tend to prefer maximal comfort, rather than prolonged survival, to which providers should adhere [41]. We also dichotomized cognitive function into that of the two severest levels, which would correspond to a CPS (Cognitive Performance Scale) level of 5 or more, and the rest [19], because, although it is difficult to predict death, intensive treatment has been shown not to prolong life in advanced dementia so that patients at this stage would be more likely to die at home [12,42].

For the cause of death, the VNS nurse was instructed to choose from the following: cancer, heart diseases, pneumonia, cerebrovascular diseases, old age and others. All causes other than "cancer" were grouped into "non-cancer" in the analysis. Other data gathered were variables related to the amount of family support, presence of family caregiver [6,21], the date when the VNS service had commenced, the use of VNS 24-hour emergency service [43], where the physician was based [9], and the use of home help services.

For the preference on the place of death, we asked where the patient and the family had respectively preferred to die: home, hospital, or unknown. "Unknown" could mean either that the patient and/or the family did have preferences which were not known by the VNS nurse, or the patient and/or the family did not have any explicit preferences [44]. The preferences were those of the latest recorded.

### Statistical analysis

Differences in the characteristics of cancer and non-cancer patients were analysed.

First, bivariate analyses were performed with chi-square test for nominal variables, the Mann-Whitney U test [45] for ordered variables and Student's *t*-test for continuous variables. Second, a logistic regression analysis of the place of death was made for cancer and non-cancer patients respectively. Patients having missing values were excluded when making the bivariate analysis. Third, a multivariate logistic regression [46], in which independent variables with p-values < 0.1 in the bivariate analysis were entered with age and gender as controlling factors, was made for the same dependent variable. Patients having missing values in ADL and cognition were excluded in the multiple logistic analysis.

For the multiple logistic regression analysis, we combined ADL and cognitive function into 4 categories: severely impaired in both ADL and cognition, severely impaired in ADL but not severely impaired in cognition, not severely impaired in ADL but severely impaired in cognition, neither severely impaired in ADL nor cognition. The grouping into these four categories was made because, after comparing two models, one having ADL and cognitive performance as independent variables, and the other as combined variables, using AIC (Akaike's information criterion)[47], goodness of fit was better for the latter (cancer: ΔAIC = -44.0; non-cancer: ΔAIC = -43.1).

For the patient's and family's preferences on the site of death the following four combinations were made: both prefer to die at home, only the patient prefers at home, only the family prefers at home, neither prefers at home (including those whose preferences were unknown).

SPSS version 16.0 was used for all statistical computations.

### Response rate and representativeness of the VNS sample

We examined the representativeness of the VNS. 1,020 of the 2,000 questionnaires sent to the VNS were returned. Four of the responses were excluded due to missing values because they had already stopped providing services, thus 1,016 VNS were analyzed (effective response rate: 50.8%). There were no significant differences in the response rates among the prefectures. When compared with the national data of the total 5,224 VNS, there were no significant differences in the mean number of total patients per month (this study: 51.3; national: 52.9, Student's *t *test p = 0.17). When the VNS were dichotomized into for-profit and non-profit, the proportion of the former was slightly lower in our sample (this study: 10.7%; national: 13.0%, Pearson's chi-square test p = 0.05).

## Results

**Figure 1:**
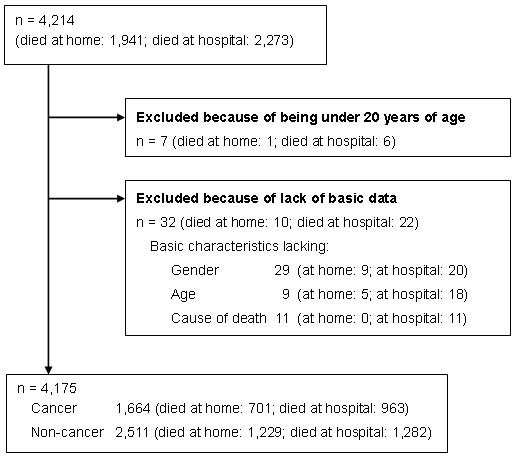
Flow of subjects analysed

After excluding 7 patients because they were under 20 years of age, and 32 patients because they lacked data on their age, gender or cause of death, a total of 4,175 deaths were reported. Of this total, 1,664 [median (range) per VNS = 1 (0-21)] were cancer and 2,511 [median (range) per VNS = 3 (1-33)] were non-cancer deaths (Figure 1). The average number per VNS was about four, which was close to the number predicted. The median percentage of cancer to all deaths per VNS was 33 (range = 0-100).

Table 1 shows the characteristics of the cancer and non-cancer patients. Of the total, cancer patients composed 39.9%, and non-cancer 60.1%. The average number of days from admission to hospital till death in patients who had been hospitalized was 9.6 (SD = 8.9) days for cancer patients, and 9.0 (SD = 9.4) days for non-cancer patients.

**Table 1:** Characteristics of cancer and non-cancer VNS patients

	Cancer		Non-cancer		
	1664		2511		p-value
	n	%	n	%	
**Cause of death**					
Cancer	1664	100			
Old age			539	21.5	
Heart disease			504	20.1	
Pneumonia			481	19.2	
Cerebrovascular disease			215	8.6	
Others			772	30.7	
**Place of death**					
Home	701	42.1	1229	48.9	< 0.001
Hospital	963	57.9	1282	51.1	
**Age at death**	76.3 ± 11.0		84.1 ± 10.0		< 0.001
**Gender**					
Male	993	59.7	1199	47.7	< 0.001
Female	671	40.3	1312	52.3	
**ADL dependence**					
J (Almost independent)	220	13.2	136	5.4	< 0.001
A (Home bound)	259	15.6	335	13.3	
B (Chair bound)	446	26.7	604	24.1	
C (Completely bedbound)	678	40.7	1388	55.3	
**Cognitive impairment**					
Independent	946	56.9	567	22.6	< 0.001
I (Almost independent)	319	19.2	536	21.3	
II (Occasional monitoring)	160	9.6	399	15.9	
III (Daily care assistance)	120	7.2	416	16.6	
IV (Continuous daily care assistance)	90	5.4	398	15.9	
M (Special professional care)	29	1.8	195	7.8	
**Patient's preference for site of death**					
Home	810	48.7	843	33.6	< 0.001
Hospital	207	12.4	145	5.8	
Unknown	647	38.9	1523	60.7	
**Family's preference for site of death**					
Home	700	42.1	1073	42.7	< 0.001
Hospital	634	38.1	578	23.0	
Unknown	330	19.8	860	34.2	
**Family caregiver**					
None/present only at night	270	16.2	483	19.2	0.013
Present at all times	1394	83.8	2028	80.8	
**Type of insurance**					
Health insurance	1052	63.2	491	19.6	< 0.001
Long term care insurance	612	36.8	2020	80.4	
**Duration of VNS services**					
25 percentile	14 days		37 days		< 0.001
50 percentile	40 days		161 days		
75 percentile	126 days		639 days		
**Use of 24-hour VNS services**					
Yes	1410	84.7	2025	80.6	< 0.001
**Where physician is based**					
Hospital	819	49.2	982	39.1	< 0.001
Clinic	812	48.8	1450	57.7	
**Use of home help services**					
Yes	547	32.9	1413	56.3	< 0.001

**ADL and cognition**					
Severely impaired in both ADL and cognition	94	5.7	510	20.3	< 0.001
Severely impaired in ADL, but not severely in cognition	584	35.1	878	35.0	
Not severely impaired in ADL, but severely in cognition	25	1.5	81	3.2	
Neither severely impaired in ADL nor in cognition	900	54.1	995	39.6	
**Patient's and family's preference for death at home**					
Both prefer at home	553	33.2	613	24.4	< 0.001
Only the patient prefers at home	260	15.6	230	9.2	
Only the family prefers at home	147	8.9	460	18.3	
Neither prefers at home	704	42.3	1208	48.1	

Missing responses have not been listed so that the total does not add to 100%.

Cancer and non-cancer patients differed significantly in their characteristics. Non-cancer patients tended to be elder, more likely to be female, more impaired in their ADL and cognitive functions, longer duration of care, and have clinic-based physicians. Home help services were not extensively used among both cancer and non-cancer patients.

Among cancer patients, although half of the patients and 42% of the families preferred to die at home, the proportion of which both had preferred was only one third. When their preferences diverged, it was the patient who had preferred to die at home. Among non-cancer patients, both had preferred dying at home in one quarter. When their preferences diverged, in contrast to cancer patients, it was the family who had preferred to die at home.

The proportion of patients whose preferences were unknown was 38.9% in the cancer group and 60.7% in the non-cancer group. Those whose preferences were not known had no significant differences in sex, age, duration of VNS services and ADL. However, those with cognitive impairment were less likely to have been able to express their preferences, and, at the two severest levels, three-quarter were not able to do so.

Table 2 shows the Odds ratio of dying at home based on a bivariate logistic regression analysis of cancer and non-cancer patients.

**Table 2:** Bivariate analysis of factors associated with dying at home

	Cancer					Non-cancer				
		
	Number dying at home	% dying at home		OR	95%CI	Number dying at home	% dying at home		OR	95%CI
**Age at death**										
for 10 years continuous^#^			**	1.17	1.07-1.28			***	1.66	1.52-1.81
**Gender**										
Male	416	41.9		1.00		520	43.4		1.00	
Female	285	42.5		1.02	0.84-1.25	709	54.0	***	1.53	1.31-1.79
**ADL dependence**										
Not severe	306	33.1		1.00		380	35.3		1.00	
Severe	368	54.3	***	2.31	1.89-2.82	830	59.8	***	2.64	2.24-3.11
**Cognitive impairment**										
Not severe	644	41.7		1.00		865	45.1		1.00	
Severe	57	47.9		1.28	0.88-1.86	364	61.4	***	1.93	1.60-2.33
**Patient's preference for site of death**										
Home	543	67.0	***	8.95	7.14-11.23	581	68.9	***	3.49	2.92-4.16
Not home (hospital/unknown)	158	18.5		1.00		648	38.8		1.00	
**Family's preference for site of death**										
Home	570	81.4	***	27.80	21.39-36.34	879	81.9	***	14.08	11.57-17.14
Not home (hospital/unknown)	131	13.6		1.00		350	24.3		1.00	
**Family caregiver**										
None/present only at night	71	26.3		1.00		218	45.1		1.00	
Present at all times	630	45.2	***	2.31	1.73-3.09	1011	49.9		1.21	0.99-1.47
**Type of insurance**										
Health insurance	456	47.4		1.00		243	48.6		1.00	
Long term care insurance	245	34.9	***	0.59	0.49-0.72	986	49.0		1.01	0.83-12.4
**Duration of VNS services**										
Shortest quartile	207	48.5		1.00		303	50.0		1.00	
2nd quartile	175	45.9		0.90	0.68-1.19	288	47.8		0.91	0.73-1.14
3rd quartile	198	49.3		1.03	0.78-1.35	297	49.3		0.97	0.77-1.21
4th quartile	119	29.4	***	0.44	0.33-0.58	333	55.1	†	1.22	0.98-1.54
**Use of 24-hour VNS services**										
Yes	592	42.0		0.96	0.73-1.26	1008	49.8	†	1.18	0.97-1.45
No	109	42.9		1.00		221	45.5		1.00	
**Where physician is based**										
Hospital	222	27.1		1.00		319	32.5		1.00	
Clinic	465	57.3	***	3.60	2.92-4.43	867	59.8	***	3.09	2.61-3.66
**Use of home help services**										
Yes	226	41.3		0.95	0.77-1.17	710	50.2		1.13	0.96-1.32
No	475	42.5		1.00		519	47.3		1.00	

Missing responses have been excluded from the total in each item.

^#^The Odds ratio shown is for 10 years increases (i.e., the tenth power of Odds ratio per one year of age).

†p < 0.1, *p < 0.05, **p < 0.01, *** p < 0.001

Factors related to dying at home in both cancer and non-cancer patients were the following: old age, severe impairment in ADL, both the patient and family preferred to die at home, and physicians based in clinics. The factor related only to cancer patients were the presence of family caregiver. The factors related only to non-cancer patients were the following: female, severe cognitive impairment, and use of the VNS 24-hour emergency service. Being in the longest quartile of VNS services (126 days for cancer; 639 days for non-cancer patients) had a significant negative effect on dying at home for cancer patients, but a modest positive effect for non-cancer patients.

Table 3 shows the Odds ratio and 95% confidence interval of dying at home in the stepwise regression model. Age and gender were not significant controlling factors in the cancer patients, but age was significant in the non-cancer patients. Cancer patients with severe impairment in ADL, but not in cognition, had a significantly higher Odds ratio for dying at home [OR (95%CI) = 1.47 (1.07-2.03)]. Non-cancer patients with severe impairments in both ADL and cognition [OR (95%CI) = 1.70 (1.27-2.27)] were more likely to die at home than those with severe impairment only in ADL [OR (95%CI) = 1.36 (1.07-1.72)].

**Table 3:** Multiple logistic regression analysis of factors associated with dying at home

	Cancer			Non-cancer		
		
	OR	95%CI	p-value	OR	95%CI	p-value
Age (for 10 years continuous)^#^	1.04	0.99-1.02	0.620	1.21	1.08-1.34	0.001
						
Gender (ref.= male)	0.87	0.64-1.18	0.363	1.18	0.96-1.45	0.112
						
**ADL and cognition (ref.= neither severely impaired in ADL nor in cognition)**						
Severely impaired in both ADL and cognition	1.50	0.79-2.82	0.216	1.70	1.27-2.27	<0.001
Severely impaired in ADL, but not severely in cognition	1.47	1.07-2.03	0.018	1.36	1.07-1.72	0.010
Not severely impaired in ADL, but severely in cognition	1.05	0.35-3.16	0.933	0.92	0.51-1.64	0.919
						
**Patient's and family's preference for death at home (ref.= neither prefers at home)**						
Both prefer at home	57.00	38.79-83.76	<0.001	12.33	9.51-15.99	<0.001
Only the patient prefers at home	4.69	3.11-7.07	<0.001	2.04	1.48-2.80	<0.001
Only the family prefers at home	20.07	12.24-32.91	<0.001	11.51	8.56-15.99	<0.001
						
**Duration of VNS services (ref.= shortest quartile)**						
2nd quartile	0.76	0.50-1.15	0.193	(Not selected by stepwise procedure)		
3rd quartile	0.75	0.50-1.13	0.170			
4th quartile	0.32	0.21-0.49	<0.001			
						
**Where physician is based (ref.= hospital)**						
Clinic	2.68	1.98-3.62	<0.001	1.99	1.62-2.45	<0.001

#The Odds ratio shown is for 10 years increases (i.e., the tenth power of Odds ratio per one year of age).

The highest Odds ratio of dying at home in cancer patients was the preferences of death at home both by the patient and the family [OR (95%CI) = 57.00 (38.79-83.76)], followed by preference of death at home only by their families [OR (95%CI) = 20.07 (12.24-32.91)]. Preference by only the patient [OR (95%CI) = 4.69 (3.11-7.07)] showed a lower Odds ratio of dying at home when compared with these two groups. In non-cancer-patients, the pattern was similar: the highest Odds ratio of dying at home was the preferences of death at home both by patient and the family [OR (95%CI) = 12.33 (9.51-15.99)], followed by only their families [OR (95%CI) = 11.51 (8.56-15.99)]. Preference by only the patient [OR (95%CI) = 2.04 (1.48-2.80)] showed a lower Odds ratio of dying at home when compared with these two groups.

Patients served by physician-based clinics were more likely to die at home, and the probability was greater for cancer patients. The longest quartile group in the duration of care was less likely to die at home than the other groups of cancer patients, but no association was observed between the duration of care and the probability of death at home in non-cancer patients.

The results of the model (-2 log-likelihood, degree of freedom of chi-square statistics, and overall rate of correct classification) were (1162.9, 12, and 84.4%) in cancer patients, and (2370.1, 9, and 78.4%) in non-cancer patients.

## Discussion

Among patients receiving VNS services who had died, there were more non-cancer patients than cancer patients. This composition differs from that in Canada, where 82.1% of the patients who died using home nursing service had been diagnosed with cancer [6]. The majority of non-cancer patients had been receiving VNS services for more than five months, with the longest being more than ten years, which reflects the fact that their original purpose had been to provide long-term care. Whether the VNS can expand their role in end-of-life care remains to be seen but their base-line position before the policy initiative did not provide validating evidence. In non-cancer patients, the length of VNS services they had received was not related to dying at home or in hospital [12], while for cancer patients it led to a higher likelihood of dying at hospital. This could be due to the fact that cancer patients were being cared for by families on the assumption that the period requiring care would be relatively short [48].

Regarding preferences on the site of death, our study showed that when the patient's and the family's preference diverged, the family's preference had a greater impact than that of the patient for both cancer and non-cancer patients. In particular, among cancer patients, when both preferred to die at home, it increased the Odds ratio by more than ten times when compared with only the patient expressing such wish. There is a caveat in that the proportion recorded as "unknown" for the patient was relatively high at 38.9% in cancer, and very high at 60.7% in non-cancer patients. There are three reasons why the proportion of "unknown" was higher for the patient. The first is that, as has been already stated, the patient may not have any explicit preference [44]. The second is that preferences on death are seldom discussed in detail within the family in Japan so that patient's preference would not be known by the family [26]. The third is that the patient's cognitive function may have declined to a level that would make it difficult to express their preferences. In non-cancer patients, 24% had severe dementia among which preference could not be elucidated from three-quarters. The future goal would be to make every effort to elicit the preference of the patient at an early stage, and also of the family at all times. When doing so, the patient's preference should be supported by relieving family's concerns [23,49]. Since the issue is difficult to discuss, a professional is needed to establish relationship with them to perform the task [50,51]. If the family could be informed of the low possibility of an effective treatment, they may prefer not to hospitalize [52].

There are two factors other than the preferences of the patient and family which had lower, but still statistically significant effects, on the site of death. First, patients who had severe impairment in ADL but not in cognition, tended to die at home in both groups [5,9,21]. Among non-cancer patients, the Odds ratio was higher for those who had severe impairment in both. Thus, appropriate protocols for the families and health care professionals to make end-of-life decisions should be designed for these patients [12,13,17]. Such protocols would be particularly needed in Japan because the patient is less likely to have communicated his or her preferences to family members [26].

Second is where the physician is based: in clinics or hospitals. Being clinic-based increased the possibility of dying at home. The choice of the physician will depend on their willingness to make home visits in the community [9,14]. In Japan, although 89% of the home visits were made by clinic-based physicians, 11% were made by hospital-based physicians [53]. This percentage may be higher for complex cases, as evidenced by the fact that hospital-based physicians comprised 49.2% of the total in cancer, which was higher than 39.1% in non-cancer. Whether the physician being hospital-based is related to the patient dying at home after controlling for medical complexity must be explored in the future.

### Limitations

There were several limitations in our study. First, the subjects were limited to patients served by VNS who had died either at home or hospital. The rest may have received care only from physicians or have died without receiving any services from health care professionals. Thus, our subjects were composed of those who may have been more conscious of their health care needs, or who are attended by physicians more aware of the benefits of VNS services. Further studies should be made of those dying at home who had not been receiving VNS services. Among those who had died in hospital, we do not know how representative our sample was because there have been no studies on the proportion of patients who had been discharged, received VNS services, and then subsequently readmitted. There is a general impression that this proportion is not high [54] implying that our sample would have unique characteristics which should be investigated in the future. The sample excluded those who may have died in hospital after four weeks of terminating VNS services. However, of those who had died within four weeks, virtually all would have been captured because the VNS must give reports on the patients' status to the physician every calendar month in order to receive their orders for the next month.

Second, since this study was designed as a retrospective, cross-sectional survey based on the VNS records, there are questions on reliability. Also, items were limited to those that could be uniformly found in the records. Data such as the patient's symptoms [55] and the family's health status [6] were not collected. Another issue is whether the patient's condition recorded reflects that when he or she died. However, there were no differences in the period from the date of the condition recorded till the date of death between those who had died at home, and those who had died in hospital: for the former, it was 14.1 days for cancer patients, and 15.1 days for non-cancer patients; for the latter, it was 23.6 days for cancer patients, and 23.7 days for non-cancer patients. Although changes in day to day condition are not recorded, since the greater majority of our subjects are over 70 years old, the extent of change in the last one month of life might not be extensive [56]. To clarify these points, a prospective study or an in-depth interview survey of a small number of patients and their families should be made in the future.

The third issue is the response rate. Although half is not necessary low for surveys made in Japan [26], we do not know about the patients who had received services from VNS that had not responded. 88% of the VNS that have responded replied that "they would like to support dying at home in their community", implying that the patients had received services from VNS more active in end-of-life care.

## Conclusions

Among those who had received end-of-life care from VNS, the proportion of non-cancer patients was greater than cancer patients. Non-cancer patients tended to be older and more impaired, and had a longer duration of care. In both groups, the greatest likelihood of dying at home was when both the patient and the family preferred, followed by only the family preferred, and then by when only the patient preferred. Health care professionals should provide opportunities for patient and the family to voice their preference on where they would wish to die, following which they should then take appropriate measures to achieve this outcome where possible.

## Competing interests

The authors declare that they have no competing interests.

## Authors' contributions

SI was involved in study conception and design, entry, analysis, revision, editing and manuscript writing. NI was involved in study conception, manuscript writing, revision, editing and overall supervision. All authors have read and approved the final manuscript.

## Pre-publication history

The pre-publication history for this paper can be accessed here:

http://www.biomedcentral.com/1472-684X/10/3/prepub

## Supplementary Material

Additional file 1 Questionnaire Click here for file
