# Supplementary material for: Predictors of dying at home for patients receiving nursing services in Japan: A retrospective study comparing cancer and non-cancer deaths
Source: BMC Palliat Care. 2011 Mar 3;10:3. doi: 10.1186/1472-684X-10-3 (PMC3061952; doi:10.1186/1472-684X-10-3)
Supplement: Additional file 1 — Questionnaire [file 1472-684X-10-3-S1.DOC]

Please answer the following questions about each patient who had died at home, or who had died in a hospital within four weeks after admission to the hospital during July to December in 2004.

< NOTE >

Please transcribe the answer to the question from the patient’s nursing record.

When you answer Q6 and Q7, please refer to the physician’s order form at the beginning of the month when death occurred or when hospitalized.

| Q1. Place of death | 1. Home 2. Hospital |
| --- | --- |
| SQ1-1. Date of hospitalization  [Only the patient Q1=2] | Day Month Year  [ ] [ ] 2004 |
| Q2. Date of death | Day Month Year  [ ] [ ] 2004 |
| Q3. Gender | 1. Male 2. Female |
| Q4. Age | [ ] |
| Q5.Cause of death | 1. Cancer  2. Heart disease  3. Cerebrovascular disease  4. Pneumonia  5. Old age  6. Others |
| Q6. ADL function | 1. J 2. A 3. B 4. C |
| Q7. Cognitive function | 1. Independent 2. Ⅰ　3.Ⅱ 4. Ⅲ　5. Ⅳ 6. M |
| Q8. Patient’s preference for site of death | 1. Home 2. Hospital　 3. Unknown |
| Q9. Family’s preference for site of death | 1. Home 2. Hospital 3. Unknown |
| Q10. Family care giver | 1. Always present(day and night)  2. Not present in daytime  3. None |
| Q11. Date of VNS service had commenced | Day Month Year  [ ] [ ] [ ] |
| Q12. Type of insurance | 1. Health insurance  2. Long Term Care insurance |
| Q13. Use of 24-hour emergency service | 1. Yes 2. No |
| Q14. Use of home help services reimbursed by LTCI | 1. Yes 2. No |
| Q15. Where physician is based | 1. Clinic 2. Hospital |
